# Profile and costs of occupational accidents reported and treated at a university hospital in Pernambuco

**DOI:** 10.47626/1679-4435-2023-1243

**Published:** 2025-01-07

**Authors:** Ana Cleide da Silva Dias, Rene Elias Gonçalves, Iraneide Nascimento dos Santos, Fernanda Gabriel Torres, Caroline Moraes Pereira Morgado, Izabel Cristina Ribeiro

**Affiliations:** 1 College of Medicine, Universidade Federal do Vale do São Francisco, Petrolina, PE, Brazil; 2 Worker Safety, Universidade de Pernambuco, Ipojuca, PE, Brazil; 3 Reference Center for Occupational Health, Petrolina, PE, Brazil; 4 Municipal Secretary of Health, Juazeiro, BA, Brazil

**Keywords:** occupational health, accidents, occupational, health care costs, saúde ocupacional, acidentes de trabalho, custos de cuidados de saúde.

## Abstract

**Introduction:**

Accidents at work are considered preventable in many situations and can cause injuries to the worker and burden the public health system and social security systems.

**Objectives:**

To describe the profile and costs of occupational accidents notified and treated at a university hospital in the state of Pernambuco from 2018 to 2021.

**Methods:**

Exploratory, ex post facto, quantitative study carried out using data from the Notifiable Diseases Information System (*Sistema de Informação de Agravos de Notificação*). Descriptive and inferential analyzes and Fisher’s exact test were performed, in addition to Poisson regression.

**Results:**

There were 603 reported accidents at work, with a mean age of 38.2 years, predominance in men (82.3%), brown race (84.2%), unknown schooling (66%) and urban residence (69.2%). Most incidents occurred in 2020 (n = 185; 27.8%), in self-employed workers (39.2%), typical (55.9%), reaching upper limbs (25.4%). It was identified that being over 37 years of age raises 95% (95%CI 0.91-0.99) the prevalence of occupational accidents in men. Being self-employed increases the prevalence of this event by 92% (95%CI 0.87-0.96; p < 0.001) and not having the employment status completed 80% (95%CI 0.73-0.87; p < 0.001). Regarding the type of accident, commuting accidents increase by 1.09 times (95%CI 1.05-1.14; p < 0.001) the prevalence of occurrences in males.

**Conclusions:**

The results indicate that the search for specific strategies to prevent accidents and, in effect, preserve the health and safety of workers and reduce government spending is relevant.

## Abstract (pt)

**Introdução:**

Os acidentes de trabalho são considerados evitáveis em muitas situações e, quando ocorrem, podem causar lesões no trabalhador e onerar o sistema de saúde pública e os regimes de previdência social.

**Objetivos:**

Descrever o perfil e os custos dos acidentes de trabalho notificados e atendidos em um hospital universitário no estado de Pernambuco, no período de 2018 a 2021.

**Métodos:**

Estudo exploratório, *ex post facto*, de abordagem quantitativa, realizado com dados do Sistema de Informação de Agravos de Notificação. Foram realizadas análise descritiva e inferencial, utilizando o teste exato de Fisher e a regressão de Poisson.

**Resultados:**

Foram notificados 603 acidentes de trabalho, com média de idade de 38,2 anos, predominando homens (82,3%), de raça parda (84,2%), com escolaridade ignorada (66%) e residentes em área urbana (69,2%). A maioria sucedeu em 2020 (n = 185; 27,8%), envolvendo trabalhadores autônomos (39,2%), acidentes típicos (55,9%) e atingindo membros superiores (25,4%). Identificou-se que ter idade superior a 37 anos eleva em 95% (intervalo de confiança de 95% [IC95%] 0,91-0,99) a prevalência de acidentes de trabalho em homens. Ser autônomo aumenta essa prevalência em 92% (IC95% 0,87-0,96; p < 0,001), e não ter a situação laboral preenchida, em 80% (IC95% 0,73-0,87; p < 0,001). Quanto ao tipo de acidente, os acidentes de trajeto elevam em 1,09 vezes (IC95% 1,05-1,14; p < 0,001) a prevalência de ocorrências no sexo masculino.

**Conclusões:**

Os resultados evidenciam a relevância de desenvolver estratégias específicas a fim de prevenir acidentes e, com efeito, preservar a saúde e segurança do trabalhador, além de reduzir gastos governamentais.

## INTRODUCTION

Due to the significant impact of occupational accidents and diseases on the morbidity and mortality of the working population, they should be considered an important public health problem.^1^ It is estimated that these injuries cause around 500,000 lost working days each year worldwide.^2^ In Brazil, even with underreporting, it is estimated that an average of 40 million fatal work accidents have already occurred, which generates assistance expenses for workers and high costs for public coffers due to retirement subsidized by the National Social Security Institute (*Instituto Nacional do Seguro Social*) (INSS).^2^

According to the National Classification of Economic Activities, in the state of Pernambuco, a total of 39,570 occupational accidents occurred between 2018 and 2020. Of these, 20,388 were classified as typical accidents and 6,390 as commuting accidents, with the victims being mostly young men aged 20 to 39.^3^ These data do not include accidents involving informally or self-employed workers, domestic workers, or workers linked to other social security systems.

The Unified Health System’s Hospital Information System contains most information related to the hospitals in the network (either directly affiliated or contracted),^4^ which are responsible for approximately 70% of all hospitalizations in the country (excluding the private network).^5^ This system uses the Hospital Admission Authorization form to collect data on the patient’s characteristics, the cause of morbidity, the diagnosis, and hospitalization costs, thus ensuring the transfer of financial resources for each hospitalization.

When injuries occur due to occupational accidents or diseases, the Notifiable Injury Information System (*Sistema de Informação de Agravos de Notificação* - SINAN) provides support for causal explanations, helping identify the epidemiological profile of a given geographic area with respect to occupational accidents (previously called “serious occupational accidents”), occupational accidents involving exposure to biological material, work-related mental disorders, work-related cancer, occupational dermatoses, pneumoconiosis, noise-induced hearing loss, and repetitive strain injury/work-related musculoskeletal disorders.^6^

Considering that Brazil has the highest occupational accident rate worldwide, with 3 deaths occurring every 2 hours, in addition to the alarming amount of underreporting and the fact that serious occupational accidents often require hospitalization,^2^ notification is a significant indicator, especially regarding hospitalization costs and missed work. In view of this, the present study described the sociodemographic profile and the hospitalization cost of occupational accident victims treated at a university hospital in Pernambuco.

## METHODS

This exploratory, *ex post facto* study with a quantitative approach is based on secondary data from the Notifiable Injury Information System and Hospital Admission Authorization forms, including all reported cases of occupational accidents registered in a university hospital in the municipality of Petrolina, Pernambuco, Brazil between 2018 and 2020. The data were collected between September and December 2022. Records in which the “occupation” field was left blank or duplicate records were excluded.

The participating hospital is managed by the Brazilian Hospital Services Company (Ebserh) and provides 100% free services to the population. It is a reference hospital in the Interstate Health Care Network of the Middle São Francisco Valley in Pernambuco/Bahia, which covers 6 health microregions with a total population of approximately 2,068,000.^7^

Using SINAN data, we analyzed sociodemographic characteristics, such as sex, age group (18-35 years, 36-59 years, or ≥ 60 years), race (White, Black, mixed, Asian, or Indigenous), professional category, International Classification of Disease code used at hospital admission, average hospital stay (< 1 day, 1-7 days, 8-14 days, 15-21 days, 22-30 days), and fatal outcome (yes or no). Information on medical procedures, the most requested tests, and the annual cost of care provided to workers admitted to the university hospital after suffering an occupational accident were collected through the Hospital Admission Authorization form data used in the Unified Health System’s Hospital Information System, which were obtained from the Municipal Secretariat.

Data on individual workers who suffered occupational accidents during the sampling period were used only in the initial stage of the study. As soon as the Hospital Admission Authorization forms were reviewed, all information that could identify the injured workers, directly or indirectly, was excluded; only quantitative elements related to the cost of care and diagnostic procedures were used in the subsequent analysis.

The data were entered into Microsoft Excel and were subsequently exported to statistical software for analysis. Descriptive analyses (measures of central tendency and dispersion, and absolute and relative frequency) and inferential analyses (Pearson’s chi-square test, Fisher’s exact test, and robust Poisson regression) were performed. Fisher’s exact test was used when absolute frequencies < 5 were > 20%. It is also worth noting that the variables “hospitalization costs” and “length of hospital stay” were categorized for association according to their medians (381.5 and 3.0, respectively). Robust Poisson regression was weighted to identify variables associated with the prevalence of occupational accidents in men, considering the frequency (82.3%; n = 547). The final model included variables with p < 0.05.

Despite using public data from a secondary source, this study was submitted to and approved by the Research Ethics Committee of the Universidade Federal de Pernambuco, Recife Campus (decision 5,571,393).

## RESULTS

The mean worker age was 38.2 (SD, 12.2) years, and the large majority were men (n = 547; 82.3%); most were of mixed race (n = 560; 84.2%), had an unreported education level (n = 439; 66%), and resided in urban areas (n = 460; 69.2%). More accidents occurred in 2020 (n = 185; 27.8%) than any other year. The largest class of victims were self-employed workers (n = 261; 39.2%). Most accidents were classified as typical (n = 372; 55.9%); the most frequently affected body region was the arms (n = 169; 25.4%); and most accidents resulted in temporary disability (n = 520; 78.2%) and hospital discharge (n = 431; 64.8%). The average length of hospital stay was 6.2 (SD, 8.7) days, and the mean hospitalization cost was BRL 965.9 (SD, 2093.0) (Table 1).

**Table 1:** Characteristics of workers admitted to a university hospital in Petrolina, Pernambuco, Brazil between 2018 and 2021 after suffering an occupational accident

Variables	n (%)^*^
Year of accident	
2018	78 (11.7)
2019	173 (26.0)
2020	185 (27.8)
2021	167 (25.1)
Employment type	
Formally employed	198 (29.8)
Informally employed	40 (6.0)
Self-employed	261 (39.2)
Public servant	8 (1.2)
Temporary worker	6 (0.9)
Day laborer	76 (11.4)
Employer	4 (0.6)
Other	3 (0.5)
Ignored	5 (0.8)
Not filled out	2 (0.3)
Accident type	
Typical	372 (55.9)
Commuting	231 (34.7)
Affected body part	
Eyes	12 (1.8)
Head	24 (3.6)
Neck	5 (0.8)
Thorax	20 (3.0)
Abdomen	8 (1.2)
Hand	146 (22.0)
Arm	169 (25.4)
Leg	51 (7.7)
Foot	2 (0.3)
Entire body	15 (2.3)
Other	1 (0.2)
Clinical outcome	
Cured	16 (2.4)
Temporary disability	520 (78.2)
Permanent partial disability	12 (1.8)
Permanent total disability	2 (0.3)
Death due to serious occupational accident	4 (0.6)
Death due to other causes	1 (0.2)
Other	1 (0.2)
Ignored	40 (6.0)
Not filled out	7 (1.1)
Hospitalization outcome	
Discharged	431 (64.8)
Transferred to another hospital	143 (21.5)
Death	5 (0.8)
Evasion	19 (2.9)
Withdrawal from treatment	5 (0.8)
Length of hospital stay (in days)	
Minimum-maximum	0.5-72.2
Mean (standard deviation)	6.2 (8.7)
Cost	
Minimum-maximum	40.4-33,243.10
Mean (standard deviation)	965.9 (2,093)

* Valid/missing sample: 602/63.

Table 2 shows the association between sex, sociodemographic characteristics, and accident type. Among women, the majority of accidents occurred in individuals < 37 years of age (n = 40; 71.4%) of mixed race (n = 51; 91.1%), with an unreported education level (n = 45; 80.4%), and official employment (n = 35; 62.5%); most accidents were classified as the commuting type (n = 39; 69.6%), resulted in temporary disability (n = 52; 92.9%), and hospital discharge (n = 32; 57.1%).

**Table 2:** Association between sex, sociodemographic characteristics, and accident type among workers admitted to a university hospital in Petrolina, Pernambuco, Brazil between 2018 and 2021 after suffering an occupational accident

Variables	Sex		p-value
	Male n (%)	Female n (%)	
Age (years)			
≤ 37	273 (49.9)	40 (71.4)	0.002^*^
≥ 37	274 (50.1)	16 (28.6)	
Race			
White	16 (2.9)	4 (7.1)	0.293†
Black	10 (1.8)	0 (0.0)	
Mixed	509 (93.1)	51 (91.1)	
Ignored	5 (0.9)	1 (1.8)	
Not filled in	7 (1.3)	0 (0.0)	
Education			
Incomplete elementary education 1	11 (2.0)	0 (0.0)	0.083†
Complete elementary education 1	4 (0.7)	0 (0.0)	
Incomplete elementary education 2	8 (1.5)	0 (0.0)	
Complete elementary education 2	5 (0.9)	0 (0.0)	
Incomplete high school	6 (1.1)	0 (0.0)	
Complete high school	33 (6.0)	2 (3.6)	
Incomplete higher education	7 (1.3)	5 (8.9)	
Complete higher education	1 (0.2)	0 (0.0)	
Ignored	394 (72.0)	45 (80.4)	
Not filled in	78 (14.3)	4 (7.1)	
Employment type			
Formally employed	163 (29.8)	35 (62.5)	< 0.001†
Informally employed	36 (6.6)	4 (7.1)	
Self-employed	249 (45.5)	12 (21.4)	
Public servant	8 (1.5)	0 (0.0)	
Temporary worker	4 (0.7)	2 (3.6)	
Day laborer	74 (13.5)	2 (3.6)	
Employer	4 (0.7)	0 (0.0)	
Other	3 (0.5)	0 (0.0)	
Ignored	4 (0.7)	1 (1.8)	
Not filled in	2 (0.4)	0 (0.0)	
Accident type			
Typical	355 (64.9)	17 (30.4)	< 0.001^*^
Commuting	192 (35.1)	39 (69.6)	
Clinical outcome			
Cure	16 (2.9)	0 (0.0)	0.234†
Temporary disability	468 (85.6)	52 (92.9)	
Permanent partial disability	12 (2.2)	0 (0.0)	
Permanent total disability	2 (0.4)	0 (0.0)	
Death due to a serious work accident	2 (0.4)	2 (3.6)	
Death due to other causes	1 (0.2)	0 (0.0)	
Other	1 (0.2)	0 (0.0)	
Ignored	38 (6.9)	2 (3.6)	
Not filled in	7 (1.3)	0 (0.0)	
Hospitalization outcome			
Discharge	399 (72.9)	32 (57.1)	0.018†
Transfer to another hospital	122 (22.3)	21 (37.5)	
Death	3 (0.5)	2 (3.6)	
Evasion	18 (3.3)	1 (1.8)	
Withdrawal from treatment	5 (0.9)	0 (0.0)	

* Pearson’s chi-square test.

† Fisher’s exact test.

Among men, occupational accidents were more prevalent among individuals > 37 years of age (n = 274; 50.1%), of mixed race (n = 509; 93.1%), with an unreported education level (n = 394; 72%) and self-employed (n = 249; 45.5%). The majority of the accidents were classified as typical (n = 355; 64.9%), resulted in temporary disability (n = 468; 85.6%), and hospital discharge (n = 399; 72.9%). In addition to this information, there was a significant difference in the variables age (p = 0.002), employment type (p < 0.001), accident type (p < 0.001), and hospitalization outcome (p = 0.018).

According to Table 3, hospitalization costs > BRL 381.47 were significantly associated with commuting accidents (57.8%; n = 118; p = 0.004), deaths (80%; n = 4; p < 0.001), and hospitalization > 3 days (59.8%; n = 159; p < 0.001).

**Table 3:** Association between medical costs and accident characteristics among workers admitted to a university hospital in Petrolina, Pernambuco, Brazil between 2018 and 2021 after suffering an occupational accident

Variables	Hospitalization costs		p-value
	≤ BRL 381.50 n (%)	≥ BRL 381.50 n (%)	
Accident type			
Typical	181 (55.0)	148 (45.0)	0.004^*^
Commuting	86 (42.2)	118 (57.8)	
Clinical outcome			
Cure	6 (46.2)	7 (53.8)	0.892†
Temporary disability	233 (50.5)	228 (49.5)	
Permanent partial disability	7 (63.6)	4 (36.4)	
Permanent total disability	1 (50.0)	1 (50.0)	
Death due to a serious work accident	1 (25.0)	3 (75.0)	
Death due to other causes	0 (0.0)	1 (100.0)	
Other	1 (100.0)	0 (0.0)	
Ignored	15 (44.1)	19 (55.9)	
Not filled in	3 (50.0)	3 (50.0)	
Hospitalization outcome			
Discharge	173 (44.2)	218 (55.8)	< 0.001†
Transfer to another hospital	82 (71.3)	33 (28.7)	
Death	1 (20.0)	4 (80.0)	
Evasion	10 (52.6)	9 (47.4)	
Withdrawal from treatment	1 (33.3)	2 (66.7)	
Length of stay (days)			
≤ 3	159 (59.8)	107 (40.2)	< 0.001^*^
≥ 3	107 (40.2)	159 (59.8)	

* Pearson’s chi-square test.

† Fisher’s exact test.

According to the multivariate analysis, the accident prevalence was 95% higher among men aged > 37 years (95%CI 0.91-0.99). Certain employment types increased the accident prevalence: it was 92% higher among the self-employed (95%CI 0.87-0.96; p < 0.001), 85% higher among public servants (95%CI 0.80-0.91; p < 0.001), 89% higher among day laborers (95%CI 0.85-0.94; p < 0.001), 89% among employers (95%CI 0.83-0.96; p = 0.002), 83% higher among those in other employment situations (95%CI 0.74-0.93; p = 0.002), and 80% higher among those who did not report their employment type (95%CI 0.73-0.87; p < 0.001).

Regarding accident type, the prevalence ratio of commuting accidents among men was 1.09 (95%CI 1.05-1.14; p < 0.001). Transfer to another hospital was also more frequent among commuting accidents (95%CI 1.01-1.13; p = 0.013) (Table 4).

**Table 4:** Characteristics associated with the prevalence of occupational accidents among men admitted to a university hospital in Petrolina, Pernambuco, Brazil between 2018 and 2021 after suffering an occupational accident

	PR	95%CI	p-value
Age (years)			
≤ 37	1	-	-
≥ 37	0.95	0.91-0.99	0.010
Employment type			
Formally employed	1	-	-
Informally employed	0.95	0.87-1.04	0.241
Self-employed	0.92	0.87-0.96	< 0.001
Public servant	0.85	0.80-0.91	< 0.001
Temporary worker	1.10	0.82-1.48	0.510
Day laborer	0.89	0.85-0.94	< 0.001
Employer	0.89	0.83-0.96	0.002
Other	0.83	0.74-0.93	0.002
Ignored	1.07	0.80-1.43	0.647
Not filled in	0.80	0.73-0.87	< 0.001
Accident type			
Typical	1	-	-
Commuting	1.09	1.05-1.14	< 0.001
Hospitalization outcome			
Discharge	1	-	-
Transfer to another hospital	1.07	1.01-1.13	0.013
Death	1.31	0.93-1.86	0.123
Evasion	0.99	0.89-1.10	0.849
Withdrawal from treatment	0.92	0.83-1.03	0.147

PR = prevalence ratio; 95%CI = 95% confidence interval.

## DISCUSSION

The results of this study provide insight into the profile and costs of occupational accident victims treated at a university hospital in Pernambuco between 2018 and 2021. Hospitalizations were more frequent in 2020, and injuries resulting in hospitalization mainly affected the arms.

The 2020 data stood out because they diverged from those of the *Social Security Statistical Yearbook* due to the COVID-19 pandemic, which significantly affected all aspects of the population, including occupational accidents. According to the yearbook, the total number of typical accidents was reduced by 16.4% (375,300 in 2019 vs 313,575 in 2020), and commuting accidents were reduced by 41.9% (102,405 in 2019 vs 59,520 in 2020).^8^

The results for affected body area differ from those of the Occupational Health and Safety Observatory.^9^ According to SINAN, 22.5% of occupational accidents involve the legs and 15.7% involve the arms. A survey of 569 occupational accidents reported that 40.6% occurred in the arms, including more serious damage to the hands, which was attributed to handling sharp objects.^10^ These data indicate the need for broader studies to identify the occupational categories most affected by accidents.

The higher number of accidents and hospitalizations among self-employed workers in 2020 may be explained by the upward trend in self-employment during the pandemic, regardless of registration as a legal entity. Many found this to be a means of remaining in the job market; self-employment reached its highest historical level in the third quarter of 2021. This is the result of government incentives to boost the economy during the pandemic, improving the business environment and reducing the bureaucracy involved in opening a business, in addition to changes in entrepreneurship culture that created more favorable conditions for starting a company.^11^

The cost of hospitalization for work-related accidents averaged > BRL 381.47 in this study. The most prevalent cost-related variables were commuting accidents, deaths, and hospitalization > 3 days. Commuting accidents are often fatal in Brazil, with 3 deaths every 2 hours. This is due to precarious safety conditions, the critical state transportation systems, and a lack of traffic enforcement, especially in the Northeast region, which by 2030 will require federal intervention to improve road safety due to its high national ranking in fatal road accidents.^2^

Regarding sex and occupational accidents, the literature is clear that men are the main victims in Brazil, especially of serious accidents, since they tend to work in more dangerous conditions, placing themselves in situations of greater risk.^12^ Therefore, a more robust analysis of sex issues in relation to occupational accidents will be necessary.

In general, our results suggest that the prevalence of occupational accidents increases among men depending on age, work situation, accident type, and hospitalization outcome. In men > 37 years of age, the risk of accidents increased by 95%. Interestingly, this differed from other studies conducted in Bahia, which found the most affected male population to be aged 20-29 years,^2^ and in Paraná, which found the most affected male population to be aged 20-34 years. On a national level, however, the most affected age group is 25 to 34 years.^13^ This shows the importance of considering regional differences in work dynamics to develop more effective accident prevention measures for each age group.^14^ However, a high rate of occupational accidents among workers aged > 37 years is to be expected, since they are the most economically productive population and have the greatest workload, resulting in a higher chance of accidents.^15^

The prevalence of occupational accidents was 92% and 89% higher for self-employed workers and day laborers, respectively. This is a major concern, since real occupational accidents tend to remain hidden among the informally employed.^13^ Studies in Bahia^16^ and Paraná^13^ found that formal employment was associated with a higher number of occupational accidents, in contrast with our results. Despite the wide variety of conditions in different occupations, it can be said that workers generally face a lack of social protection, and submission to market demands can contribute to occupational accidents.^17^

Additionally, increased selfand informal employment is a result of the new work scenario worldwide, which, under the strong influence of globalization, has increased the number of occupations while simultaneously reducing worker rights.^18^ Thus, reduced job security is reflected in an increased risk of accidents, especially in the most productive age group. During the pandemic, a growing number of young people were taking risks in an uberized job model, since COVID-19 affected the number and quality of available jobs, resulting in the growth of groups vulnerable to “changes in the labor market”, i.e. those in precarious living situations, youth, women, and migrants.^19^

Another worrying finding in this study is the failure to report employment status on the SINAN form, since it is associated with an 80% higher accident prevalence. Incomplete information is a serious flaw in the reporting system, because it leads to insufficient data for companies or government agencies to develop effective preventive measures.^20^

This study found that men are 1.09 times more likely to suffer commuting accidents than typical occupational accidents. A retrospective study found that the incidence of commuting accidents increased from 88.17 to 105.88 between 2009 and 2016, which may have been influenced by the greater number of vehicles per inhabitant and, consequently, by an overall increase in traffic accidents. This is worrying, since it could affect the social security system financially through lost work due to hospitalizations or surgeries.^15^ Men may have been more affected by commuting accidents because they represent a higher percentage of workers in the job market than women.^15^

Regarding hospitalization outcome, there was a higher probability of transfer to another hospital for commuting accidents, indicating that this type of accident is more severe and is a predictor of mortality.^21^ It should also be noted that when employees miss work due to occupational accidents, the remaining staff may become overloaded with work and temporary workers may be hired, who often receive little or no training,^22^ both of which can lead to further accidents.

Regarding the profile of accident victims, although statistically significant results were not found for some sociodemographic variables, others differed significantly. Race is one example. Black workers tend to die from occupational accidents at an earlier age than Whites.^23^ Since the colonial period, institutional racism has relegated the black population to the lowest positions^24^ of manual labor and degrading working conditions.^25^

The workers’ education level was ignored in 66% of the hospitalization records, despite its importance, given that low education can make it difficult to perceive risks in the work environment, limiting adherence to preventive measures.^26^ The occurrence of occupational accidents among urban residents is mostly due to the development of self-employment and the informal labor market, which has grown significantly in Latin America due to the decline in official employment in recent years concomitant with the COVID-19 pandemic.^27^

### LIMITATIONS

This study is limited by its design, which in addition to not allowing for causal inferences, reflects the state of affairs during the study period, especially regarding the accident victims’ education level, since these questions were ignored on the majority of forms. Future studies should assess the types of occupations affected by accidents, which could provide a more comprehensive view of the possible occupational risks in the work environment.

## CONCLUSIONS

According to our results, occupational accidents may be associated with several sociodemographic characteristics. Characterizing the epidemiological profile of workers affected by occupational accidents provides an opportunity to identify the economically active population at risk and the possible consequences for its health. Provided this information, effective prevention strategies can be developed, aiming to protect the health and safety of workers and reduce government spending.
